# Complete Genome Sequences of Six BI Cluster *Streptomyces* Bacteriophages, HotFries, Moozy, Rainydai, RavenPuff, Scap1, and SenditCS

**DOI:** 10.1128/MRA.00993-18

**Published:** 2018-09-27

**Authors:** Devin Blocker, Matthew Koert, Courtney Mattson, Harsh Patel, Priya Patel, Rishit Patel, Hemanta Paudel, 2017 UMBC Phage Hunters, Ivan Erill, Steven M. Caruso

**Affiliations:** a Department of Biological Sciences, University of Maryland Baltimore County, Baltimore, Maryland, USA; Queens College

## Abstract

Six double-stranded DNA Streptomyces bacteriophages, HotFries, Moozy, RavenPuff, Scap1, Rainydai, and SenditCS, were isolated using the phytopathogen Streptomyces scabiei as a host. These phages have been identified as *Siphoviridae* and members of cluster BI by genomic analysis.

## ANNOUNCEMENT

Bacteriophages HotFries, Moozy, RavenPuff, Rainydai, SenditCS, and Scap1 were isolated by direct plating of processed soil samples collected around Baltimore, Maryland, and Kottayam, India (see Table 1), onto lawns of Streptomyces scabiei RL-34 (ATCC 49173), a phytopathogen responsible for common scab disease (1). The samples were collected and isolated by undergraduate researchers as part of the SEA-PHAGES program at the University of Maryland Baltimore County (2).

**TABLE 1:** Properties of six cluster BI *Streptomyces* phages

Phage name	Genome size (bp)	9-bp 3′ overhang sequence	% GC content	No. of ORFs^*a*^	Origin	Cluster	GenBank accession no.
HotFries	43,699	5′-CGCCGCCCT	60.9	57	Owings Mills, MD	BI2	MH155869
Moozy	43,545	5′-CGCCGCCCT	61.2	58	Olney, MD	BI2	MH155872
RavenPuff	43,709	5′-CGCCGCCCT	60.9	59	Catonsville, MD	BI2	MH155878
Scap1	43,060	5′-CGCCGCCCT	60.9	55	Kottayam, India	BI2	MF975637
Rainydai	57,623	5′-CGCCCGCCT	58.1	91	Rosedale, MD	BI4	MH155877
SenditCS	55,993	5′-CGCCCGCCT	58.2	86	Ellicott City, MD	BI4	MH155880

a ORFs, open reading frames.

The phages were plaque purified on lawns of S. scabiei grown on supplemented nutrient agar (3) with Trypticase soy soft agar overlays incubated for 24 to 48 h at 30°C, and a high-titer crude lysate was harvested as described (4). Examination by transmission electron microscopy revealed the phages to be *Siphoviridae*, with icosahedral capsids with an average width of 54 nm (standard deviation [SD], ±5 nm) and flexible tails with an average length of 254 nm (SD, ±15 nm). The host range of the phages was tested by spotting diluted crude lysate on lawns of Streptomyces spp. (5). All six phages infected Streptomyces mirabilis NRRL B-2400 and Streptomyces neyagawaensis ISP 5588 at similar efficiencies of plating (EOP). Moozy, Rainydai, and RavenPuff also infected Streptomyces azureus SC 2364 but at a reduced EOP. SenditCS, Scap1, and HotFries lysed S. azureus SC 2364 but did not produce infectious particles. RavenPuff demonstrated the broadest host range, infecting Streptomyces bobili IMRU 3310, S. bottropensis ISP-5262, S. diastatochromogenes IFO 3337, and S. griseus subsp. griseus (ATCC 10137).

Phage DNA was isolated using the Wizard DNA clean-up system (Promega). Sequencing libraries were prepared by the Pittsburgh Bacteriophage Institute from genomic DNA using an NEB Ultra II FS kit with dual-indexed barcoding. Forty-eight libraries were then pooled and run on an Illumina MiSeq instrument, yielding at least 80,000 single-end 150-base reads for each genome. These reads were then assembled using Newbler version 2.9 with default settings, and in each case, the assembled reads yielded a single phage contig which was checked for completeness, accuracy, and phage genomic termini by using Consed version 29 (6). Phage genomes averaged 2,549× coverage and were identified as linear with 9-bp 3′ sticky overhangs. Genome annotation was completed using DNA Master (7).

All six bacteriophages were classified as members of cluster BI based on nucleotide conservation, genomic synteny, and phylogenetic analysis (8). HotFries, Moozy, RavenPuff, and Scap1 are in subcluster BI2 and have an average genome length of 43,503 bp (SD, ±305 bp) and a GC content of 61.0% (SD, ±0.2%). Rainydai and SenditCS are in subcluster BI4 and have an average genome length of 56,878 bp (SD, ±1,153 bp) and a GC content of 58.2% (SD, ±0.1%). Between 55 and 59 protein-coding genes were identified in the BI2 phages, while 86 and 91 protein-coding genes were identified in the BI4 phages (see Table 1). No tRNA genes were identified. The BI phages displayed low average nucleotide identity (ANI) between each other (0.76 [SD, ±0.12]), although the subcluster ANIs ranged from 0.88 to 0.98 (9). For additional information, see the Actinobacteriophages Database (10).

### Data availability.

The GenBank accession numbers for the genome sequences reported here are provided in Table 1. The raw sequencing reads are available in the SRA under the accession number SRP159070.

## References

[1] Lerat S, Simao-Beaunoir A-M, Beaulieu C 2009 Genetic and physiological determinants of *Streptomyces scabies* pathogenicity. Mol Plant Pathol 10:579–585. doi:10.1111/j.1364-3703.2009.00561.x. 19694949 PMC6640508

[2] Jordan TC, Burnett SH, Carson S, Caruso SM, Clase K, DeJong RJ, Dennehy JJ, Denver DR, Dunbar D, Elgin SCR, Findley AM, Gissendanner CR, Golebiewska UP, Guild N, Hartzog GA, Grillo WH, Hollowell GP, Hughes LE, Johnson A, King RA, Lewis LO, Li W, Rosenzweig F, Rubin MR, Saha MS, Sandoz J, Shaffer CD, Taylor B, Temple L, Vazquez E, Ware VC, Barker LP, Bradley KW, Jacobs-Sera D, Pope WH, Russell DA, Cresawn SG, Lopatto D, Bailey CP, Hatfull GF 2014 A broadly implementable research course in phage discovery and genomics for first-year undergraduate students. mBio 5:e01051-13. doi:10.1128/mBio.01051-13. 24496795 PMC3950523

[3] Smith MCM, Hendrix RW, Dedrick R, Mitchell K, Ko C-C, Russell D, Bell E, Gregory M, Bibb MJ, Pethick F, Jacobs-Sera D, Herron P, Buttner MJ, Hatfull GF 2013 Evolutionary relationships among actinophages and a putative adaptation for growth in *Streptomyces* spp. J Bacteriol 195:4924–4935. doi:10.1128/JB.00618-13. 23995638 PMC3807479

[4] Sarkis GJ, Hatfull GF 1998 Mycobacteriophages. Methods Mol Biol 101:145–173. doi:10.1385/0-89603-471-2:145. 9921476

[5] Jacobs-Sera D, Marinelli LJ, Bowman C, Broussard GW, Guerrero Bustamante C, Boyle MM, Petrova ZO, Dedrick RM, Pope WH, Science Education Alliance Phage Hunters Advancing Genomics And Evolutionary Science Sea-Phages Program, Modlin RL, Hendrix RW, Hatfull GF 2012 On the nature of mycobacteriophage diversity and host preference. Virology 434:187–201. doi:10.1016/j.virol.2012.09.026. 23084079 PMC3518647

[6] Gordon D, Abajian C, Green P 1998 Consed: a graphical tool for sequence finishing. Genome Res 8:195–202. doi:10.1101/gr.8.3.195. 9521923

[7] Pope WH, Jacobs-Sera D 2018 Annotation of bacteriophage genome sequences using DNA Master: an overview. Methods Mol Biol 1681:217–229. doi:10.1007/978-1-4939-7343-9_16. 29134598

[8] Pope WH, Bowman CA, Russell DA, Jacobs-Sera D, Asai DJ, Cresawn SG, Jacobs WR, Hendrix RW, Lawrence JG, Hatfull GF, Science Education Alliance Phage Hunters Advancing Genomics and Evolutionary Science, Phage Hunters Integrating Research and Education, Mycobacterial Genetics Course 2015 Whole genome comparison of a large collection of mycobacteriophages reveals a continuum of phage genetic diversity. Elife 4:e06416. doi:10.7554/eLife.06416. 25919952 PMC4408529

[9] Yoon S-H, Ha S-M, Lim JM, Kwon SJ, Chun J 2017 A large-scale evaluation of algorithms to calculate average nucleotide identity. Antonie Van Leeuwenhoek 110:1281–1286. doi:10.1007/s10482-017-0844-4. 28204908

[10] Russell DA, Hatfull GF 2017 PhagesDB: the Actinobacteriophage Database. Bioinformatics 33:784–786. doi:10.1093/bioinformatics/btw711. 28365761 PMC5860397

